# Nanocomposites containing titanium dioxide for environmental remediation

**DOI:** 10.1080/15685551.2021.1876322

**Published:** 2021-01-20

**Authors:** Soad Z. Alsheheri

**Affiliations:** Department of chemistry, King Abdulaziz University, Jeddah, SA, Saudi Arabia

**Keywords:** Nanocomposites, titanium dioxide, photocatalysis, titania nanoparticles

## Abstract

Titania is considered to be one of the most versatile material in its nanoform. Scientific community looks towards it to address various pressing global problems. One such problem is aquatic pollution arising from organic chemicals such as dyes, pesticides, antibiotics etc. due to industrial, domestic and agricultural activities. Titania proves to be very effective to address this problem owing to its superior photocatalytic properties. In this review, we will review the recent advances in titania-based nanocomposites. The recent advances discussed in this review include synthesis of titania, modification of titania, exploration of various supports such as silica, carbon, graphene etc., that is documented to enhance its environmental remediation properties.

## 1. Introduction

Nanoscience changed the world we live significantly than we can imagine before the discovery/invention of this field of science [1]. Materials developed in the nano-size regime are termed as nanomaterials. Nanomaterials are sought to be very interdisciplinary in nature that overlaps the fields of nanotechnology and nanoscience. Since the early 90’s of the last century, the science of development of nanomaterials was very steep and led to numerous publications. Sophistication was achieved in the development of materials with precise control in size, shape, morphology, physico-chemical properties, ability to modify the surface of nanomaterials and customization to specific needs [2–4]. This article/review primarily focuses on recent developments in the synthesis of nanoporous titania-based materials with applications in environmental remediation.

Titanium dioxide widely known as titania was mostly known for its use as white pigment in early last century [5,6]. Interest in titania to the scientific community was triggered by pioneering studies from Honda and Fujishima who used this material for water splitting with use of UV radiation [7]. This study was further explored by Schrauzer et al who reported the photocatalytic water decomposition using titania impregnated with Pt and/or Rh in catalytic amounts [8]. Those pioneering studies led to deep investigation of titania for a variety of applications ranging from photocatalytic hydrogen evolution [9,10], removal of organic pollutants [11–13], sunscreens [14], synthesis of polymers [15], bio-fuel production [16,17], development of dye-sensitized solar cells [18], catalysis [19,20], sensors [21], drug-delivery [22,23], to name a few. The primary attraction of titania to be able to be used in a gamut of research fields listed above stems from its properties: low-cost, benign, abundant, fair physical and chemical stability of the material [24,25].

**Figure 1.**
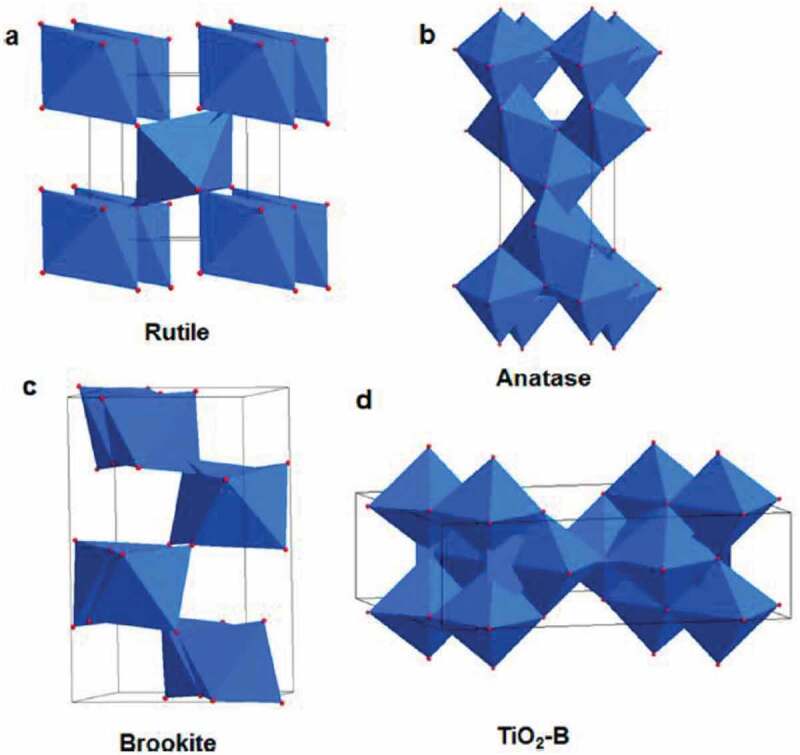
Polymorphs of Titania a) rutile b) anatase c) brookite and d) TiO_2_-B. Figure adopted from [26]

Titania the prominent transition metal oxides exists commonly in four polymorphs [27]. They are anatase, rutile, brookite and TiO_2_(B) shown in Figure 1. Rutile has a tetragonal structure and contains six atoms in a unit cell. Rutile and anatase polymorphs have chains of TiO_6_ octahedra with each Ti ^4+^ is surrounded by an octahedra made of six O^2-^ ions. These structures vary in the distortion of each octahedron and via assembly pattern of octahedra chains. Rutile has mild orthorhombic distortion, whereas anatase has severe distortion such that its symmetry is lower than orthorhombic. The interatomic distance between titanium atoms in anatase are larger than in rutile and Ti-O distances are smaller than in rutile. Further, in the rutile structure, each octahedron is in contact with 10 neighbouring octahedrons. Anatase has each octahedron in contact with eight neighbours [28,29]. This structural difference in the lattice structures lead to different mass densities and electronic band structures between the two prominent polymorphs of titania. Anatase has a band gap of 3.2 eV for anatase and 3.0 eV for rutile. Rutile is thermodynamically the more stable form. Of the various gpolymorphs and crystal structures, everything can be converted to rutile form when they are subjected to high temperatures. Brookite is a less common phase of titania that is difficult to synthesize in a laboratory. Similar to brookite, TiO_2_-B is also less investigated for photocatalytic applications. The major characteristics of four polymorphic forms of titania is listed in Table 1.Thus, the majority of studies in titania pertaining to synthesis and photocatalytic activity is based on anatase and rutile. Degussa P 25 is the commercial titania nanomaterial with approximate surface area of 50 m^2^/g, and the crystallite size is 30 nm with mixed phases of rutile and anatase in the ratio of 3:7.

**Table 1.** Physical properties of anatase, rutile, brookite and TiO_2_-B

	Anatase	Rutile	Brookite	TiO_2_-B
Theoretical Density (g/cm^3^)	3.84	4.26	4.11	3.64
Unit Cell Volume (Å^3^)	34.02	31.12	32.2	35.27
Band Gap (eV)	3.2	3.0	3.3	3.29
Space group	*P42/mnm*	*I4I/amd*	Pbca	C2/m
Molecule/cell	4	*2*	8	1
Crystal structure	Tetragonal	*Tetragonal*	orthorhombic	Monoclinic

## 2. Mechanism of photocatalysis

Several studies were pursued to understand the pathways/process of semiconductor photocatalysis to enhance the quantum efficiency and extend the absorption wavelength of titania catalyst. Understanding this crucial step will allow us to use the photocatalysis at industrial scale level.

**Figure 2.**
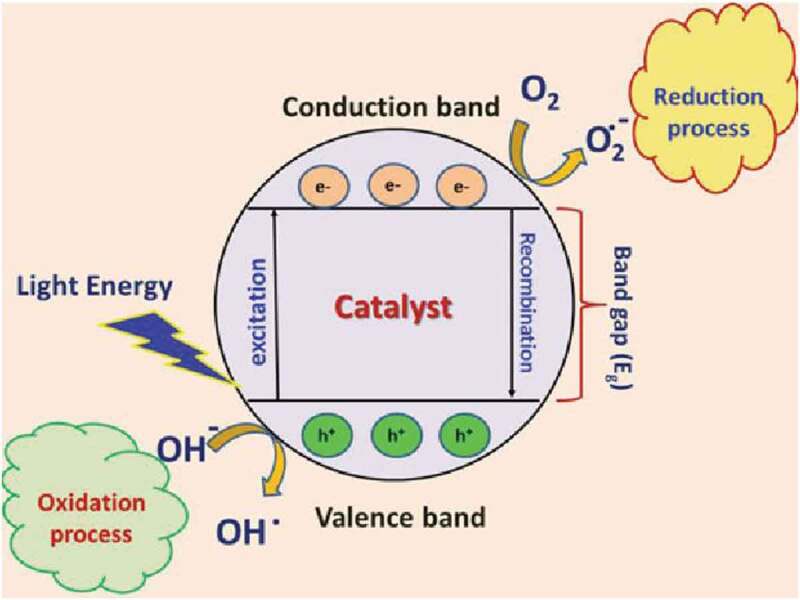
Graphic mechanism of photocatalysis [30]

First step in photocatalysis is the formation of electron-hole pairs in the titania semiconductor via excitation of light as shown in Figure 2. When the titania was subjected to sufficiently energetic light, electrons and holes are produced in conduction and valence bond, respectively. Such formed electron and hole may recombine with each other in the volume or on the surface of the semiconductor particle and dissipates its energy as heat. Electrons that are capable of reaching to the surface of photocatalyst are considered good oxidants and similarly the hold are good reductants. The electron-hole recombination is pivotal that occurs in volume or surface dampens the charge transfer process of catalyst, thereby, dampens the photocatalytic efficiency of the reaction. This electron-hole recombination can be suppressed if the electron acceptor and donor species are adsorbed on the surface of the semiconductor surface.

The photocatalytic efficiency of a reaction is dependent on the size of the titania semiconductor particles. As the size of catalyst particle drops, the active sites increase, on the contrary, if it becomes too small the recombination process may dominate, hence, an optimal size is necessary to find a balance. The timescale for generation of electron-hole pair was calculated as 100 fs through laser flash photolysis experiment [31,32] Such generated electron-holes will be attached to the surface of titania in 100 ps to 10 ns. The hydroxyl radicals attached to surface of titania takes 10 ns [33] for its formation, meanwhile, the recombination of electron-hole takes 10–100 ns [34]. 100 μs to ms are necessary for transfer of electron to oxygen molecules during the charge transfer process. Thus, the quantum efficiency for charge transfer is dependent on two competing reactions 1) competition between charge carrier recombination (100–10 ns) and trapping (100 ps to 10 ns) and 2) competition between trapped carrier recombination and interfacial charge transfer. The lifespan of electron and hole can be increased by charge carrier trapping [35]. Defects in the surface of catalyst improve charge carrier trapping. Conduction band electrons are trapped typically in nanoseconds range and valence band electrons trapping requires around 250 ns [36]. ESR studies has revealed the existence of trapped photogenerated electrons through the presence of Ti^3+^ sites. Trapped holes are of two types: shallow and deep. Deeply trapped holes are considered to be unreactive and long-lived whereas shallowly trapped holes are very reactive and exist in thermal equilibrium with free holes.

Electron-hold recombination in titania nanomaterial can be suppressed by induction of other transition metals in catalytic amounts. Further, introduction of transition metals facilitates charge transfer process between the semiconductor and solution interface [37]. Doping of titania with transition metals causes defect sites, which in turn helps trapping of holes in the valence band and electrons in the conduction band. Additional benefit of doping in titania causes visible light adsorption through defects created in the band gap. If titania doped with two different transition metals, it was observed to improve the photoinduced charge separation through synergistic action of dual doping [38].

Beyond recombination, electrons and holes also react with oxygen and water to create a variety of oxygen species in the reaction. Nature of oxygen species vary depending on chemical reaction, regardless, the prominent oxygen species are superoxide radical, hydrogen peroxide radical and hydroxyl radical are important for degradation and mineralization of organic compounds [39]. Superoxide radical is believed to be important in mineralization process and hydroxyl and hydrogen peroxide contribute to degradation of organic pollutants. Particularly, hydrogen peroxide’s contribution to degradation of organic pollutants is via acting as an electron acceptor or behaving as a source of hydroxyl radical. An alternate pathway that was proposed by certain researchers are direct hole transfer to organics [40]. In some reactions, both pathways were found to happen. The pathway for degradation of organic compounds depends on the structure of organic compound and adsorption ability of titania in its surface. When titania has poor adsorption capability, they oxidized through hydroxyl radical pathway. Alternatively, if titania has good adsorption power, then the organic compound will be decomposed by direct hold oxidation pathway.

The primary limitation of use of titania as photocatalyst arises due to its short wavelength absorption in the UV region, thus, limited quantum efficiency. To improve the light absorption of photocatalyst from UV region through visible region required modification of catalyst with doping of nonmetal and metallic elements. The dopants reported were non-metals N, C, S, B, F, Cl, Br and metals Cr, Ce, Co, V, Fe, Ni, and W [41–45]. Those dopants (metals) were found to increase the absorption in visible regions through charge transfer transition of d electron from metal dopant to conduction band or valence band of titania. It is demonstrated that transition metal induction in titania catalyst at an optimal level suppresses the recombination rate. The enhancement in visible light absorption of titania through non-metal doping is not clearly understood to date [46,47]. Another strategy to extend the light absorption of titania was through introduction of materials such as sensitizers that has narrow band gap. The sensitizer range from noble metals, inorganic semiconductors and organic dyes. Examples to name a few are CdS, CdSe, PbS, Bi_2_S_3_, Ag_2_S and Sb_2_S_3_, Ag, Au, Pt etc [48,49]. Other sensitizers are organic dyes and transition metal complexes such as phthalocyanine, metallophorphyrins, polypyridine complexes [50,51].

## 3. Synthesis of titania nanoparticles

The ability to control physico-chemical properties such as size, shape, morphology, composition of phases is critical to photocatalytic efficiency. The ability to control those properties often arise from its synthesis strategies. Strategies to synthesize titania and titania nanomaterials are as diverse as its applications. The conventional synthesis methods are sol-gel, solvothermal, hydrothermal, chemical vapour deposition, direct oxidation, electrodeposition, sonochemical and microwave. We will present a brief discussion of each of those synthetic methods below, and all of those synthesis methods produce titania in anatase or rutile form primarily as they are the ones found to be active for photocatalysis.

### 3.1. Sol-Gel method

Sol-gel method is the most adopted to synthesize structures that are porous, unique in shape such as thin fibers to dense powders and films. In this process, a liquid solution, a sol, which is formed by hydrolysis of the precursor colloidal particles in the liquid is converted to solid phase gel [52,53]. In other words, it is a stable dispersion of colloidal particles in a suitable solvent. The solid phase gel was constructed via agglomeration of colloidal particles. Alternatively, gel can be formed from linkage of polymeric chains as well. The gel once formed cannot be deconstructed as it is an irreversible process. The interaction between gel particles is typically covalent in nature. In a general synthesis of titania via sol-gel process, titania was formed by hydrolysis first and followed by condensation reaction of titanium alkoxide [54,55]. The characteristics of titania is hugely dependent on the rates of hydrolysis and condensation. The reaction parameters that affect the rate of hydrolysis and condensation are pH of reaction medium, solvent type, additives, reaction temperature, reactivity of metal alkoxide, water to alkoxide ratio etc [56,57]. The titania particles synthesized through this route typically have nano-dimensions. Further, the sol-gel process is the most widely adopted route to synthesize titania as it involves simple equipment, low temperature, generates highly homogeneous and pure products and allows for modification of surface and synthesis procedures. The titania synthesized via this route have high specific surface area, allows the manipulation of shape, size, distribution, porosity and introduction of dopants in the structure of catalyst at low temperature [58–62].

### 3.2. Sol method

This method involves titanium chloride reaction with oxygen donors such as ethers or metal alkoxides. This synthesis path is non-hydrolytic in nature. The titanium chloride and titanium alkoxide reacts to form Ti-O-Ti bonds/bridges [54,63,64]. The alkoxides are generally formed by reaction of titanium chloride with alcohols or ethers. In this reaction, the length of the carbon chain in alcohol affects the reaction speed.

stabilizes the nanocrystals and offer ready exchange of surface capping groups and therefore

The reactions usually require elevated temperatures in anhydrous organic solvents forming nanocrystals that has no hydroxyl group on the surface. Control over the surface can be achieved by help of coordination chemistry which stabilizes the nanocrystals and offer ready exchange of surface capping groups and therefore control properties of nanocrystals obtained. The halogens coupled with titanium dictates the particle size. In addition, the shape and size of titania particles are influenced by addition of surfactant. Depending on the surfactant used, titania obtained can be diamond-shape, bullet shape, nanorods with or without branching [65–67].

### 3.3. Hydrothermal method

Hydrothermal synthesis of titania involves autoclaves with or without teflon liners, the reaction mixture will be subjected to controlled temperature. The volume of solution used and temperature determines the actual pressure inside the container. The typical temperature used in the process ranges from 373 K to 873 K. This process has good control on synthesis of titania in anatase phase. At temperatures above 573 K, anatase phase is observed exclusively whereas, at a temperature below 573 K, a mixture of anatase and brookite was observed due to epitaxial growth mechanism. Hydrothermal is assumed to be environmentally benign and proper control of temperature allows to synthesize titania in the desired crystallite size and form, specific surface area, morphology, phase, size distribution and superior interfacial properties [54,68,69].

### 3.4. Solvothermal method

**Figure 3.**
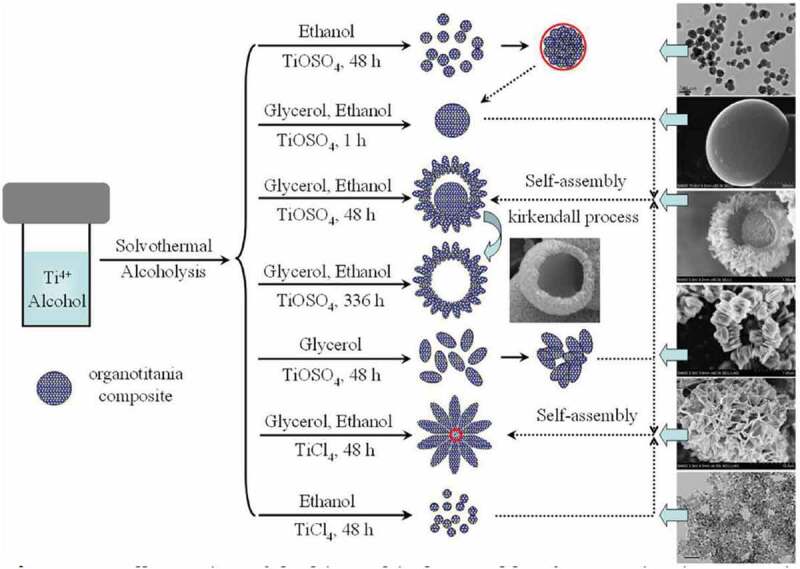
**Effect of reaction conditions on solvothermal synthesis of titania** [75]

This method can be viewed as a subset of hydrothermal method as it is identical in all respects of hydrothermal synthesis except for the fact that solvents other than water can be used. Because these solvents can have higher boiling points than water, the temperature for solvothermal process can be extended beyond what can be achieved through water. This process also offers the opportunity to control the size, shape and crystallinity of titania nanoparticles as shown in Figure 3 [70,71]. Further, this route may facilitate the synthesis of titania nanoparticles as spheres, rods, wires with or without the use of surfactants [72–74]. Solvents that have different physical and chemical properties affect the solubility, reactivity, diffusion behaviour of reactants hence the morphology and crystallization of titania were affected.

### 3.5. Chemical vapour deposition (CVD)

In CVD, the reactants were vaporized and condensed to obtain a solid-phase material. Generally, this procedure is used for coating materials to manipulate the thermal, electrical, mechanical, optical and corrosion resistance on substrates. This process is typically conducted in a vacuum chamber, the heat supplied is used to heat the gases in chamber and facilitates the deposition reaction, in an inert atmosphere or in the presence of a gas. Wu et. al. reported fabrication of the aligned TiO2 nanorods as well as TiO2 nanowalls by a catalyst-free template-free, MOCVD technique. Structural analyses suggest that single-crystalline rutile and anatase TiO_2_ nanorods were constructed at high temperature, 630 °C and 560 °C, respectively, while anatase TiO2 nanowalls formed at 535 °C.

Synthesis of the well-

aligned TiO_2_ nanorods was suggested to relate to the relative growth rate of a variety of crystal

faces bounding the tetragonal TiO_2_ nanocrystal [76]. Through suitable manipulation of

temperature and pressure, the phases and morphology of titania can be manipulated [76]. Many

processes were used and fall under CVD technique, such as electrostatic spray hydrolysis, which

was used to prepared anatase (TiO_2_) nanoparticles that were unimodal. The method involves

spraying the precursor solution at low Ti concentration in a ‘multi-jet’ mode. In order to avoid

coagulation of solidifying particles, the charged aerosol was heated first. Two competing

processes were suggested to occur: disruption of droplets into smaller droplets, and development

of a hard outer shell, which stopped the partially dried droplets from further disruption [77].

Another process of CVD is thermal plasma pyrolysis [78,79]. The method of thermal plasma

pyrolysis involves using TiC micron powders which can be efficiently oxidized in the Ar-

O_2_ plasma and the degree of oxidation can be controlled by the oxygen feedback in the plasma

gases. The TiO_2_ particles produced by this process consist of well-defined mono-dispersed

TiO_2_ micron spheres and nanosize powders [79]. Ultrasonic spray pyrolysis is also a method of

CVD and was used to obtain multiwallednanoparticles, the mechanism of the formation of the

nanoparticles were suggested to involve harmonisation of the ultrasonic initiator and induced

oscillating field of the aerosol drop itself as suggested by theoretical studies [80]. Nanosized

titanium dioxide (TiO_2_) was also produced by using laser-induced pyrolysis technique utilizing

titanium isopropoxide as a liquid precursor [81]. The use of ultrasonic-assisted hydrolysis

process during the synthesis of nanomaterials could be beneficial to enable the growth of smaller

homogeneous nanoparticles and lead to an increase in surface area and diminish crystallite

growth [82].

### 3.6. Direct oxidation method

In this method, typically titanium metal is oxidized using hydrogen peroxide or any other suitable oxidants [83]. The titanium metal plate will be pickled with HF solution, followed by ultrasonic cleaning. Thus, cleaned metal will be soaked in hydrogen peroxide solution with or without metallic salts [84,85]. The dissolution precipitation mechanism generates the crystalline TiO_2_. The crystallinity of TiO_2_ was influenced by metallic salts such as sodium chloride, sodium fluoride, sodium sulfate etc. The alternate oxidizing agents are ammonium fluoride and malonic acid in place of hydrogen peroxide. At high temperatures, acetone too oxidizes titanium to form titania nanorods. The nanorods synthesized using acetone were very dense and well aligned [86].

### 3.7. Sonochemical synthesis

This method is also widely adopted to synthesize a wide range of nanomaterials such as oxides, carbides, alloys and colloids. Ultrasound irradiation causes rapid formation, growth and collapse of unstable bubbles in liquids, where temperature can be as high as 5000 K and pressures up to 20 MPa and high cooling rates of 10^10^ Ks^−1^ [87]. Several researchers have reported synthesis of titania through this route [88–91]. Researchers have found that it is possible to synthesize titania phase selectively through manipulation of titania precursors, temperature strength of ultrasound [92,93]. Additionally, it is possible to control the shape as well [94].

### 3.8. Microwave method

Dielectric materials tend to interact with electromagnetic waves with high frequency. One such electromagnetic wave is microwaved. Microwaves have heating frequencies ranging from 900 to 2450 MHz. At low microwave frequencies, the ionic constituents of the material can transfer the energy from microwave to the material. At higher frequencies, the molecules with permanent dipole which tends to realign to absorb the energy from the microwave. Microwaves allow for quick heat transfer, volumetric and selective heating [95]. Microwaves allow to synthesis titania in as short as 5 min to 60 min, whereas, the conventional synthesis requires 60 min to 32 h and temperature up to 468 K [96]. Microwave allows synthesis of titania in the form of nanorods, nanocrystals and phase selective form of titania by tuning the microwave power [97–103].

## 4. Applications in environmental remediation

Industrial revolution, modernization, population growth all lead to aggravated use of natural resources causing non-benign and irreparable damages to our environment. Natural resources are seriously affected by industrial discharges and effluents. Water bodies are the most vulnerable of ecosystem affected by mankind due to industrialization in the past century. The common contaminants of water bodies come from a variety of sources such as chemicals, fertilizers, pesticides, batteries, dyes, etc. to name a few. The primary aquatic pollutants are organic compounds such as phenols, surfactants, pesticides, dyes, and organic halogens from the above-mentioned industries. Such organic pollutants have longevity, resistance and penetrability into the food chain and have adverse health consequences on humans and other living beings in our ecosystem. The health effects of these organic pollutants range from carcinogenic, reproductive disorders, congenital disabilities, endocrine effects, growth disorders in children and infants etc. Various organic pollutants dye and pesticides pose a major threat to humans and aquatic life due to its large-scale release in water bodies. Thus, the contamination causes water scarcity as it cannot find use in domestic and agriculture purposes. Thus, it is imperative to improve the water quality through remediation to use it for day-to-day needs. Thus, it is very important to develop wastewater remediation strategies that are cost-effective and environmentally benign. Currently various methods have been investigated by the scientific community for wastewater remediation in the past few decades.

The strategies for remediation can be broadly classified as biological, physical and chemical methods. Of these various remediation processes chemical methods are of interest to us. The commonly used chemical strategies are advanced oxidation processes (AOP), ozonation, electrochemical methods, and photocatalytic.

### 4.1 Advanced oxidation process

It is a water remediation technique for removal of organic pollutants, bio-recalcitrant and pathogens. This process involves generation of highly reactive chemical species that can convert the pollutants to biodegradable substances. Most AOP’s involve generation of hydroxyl radical which is very powerful to oxidize and mineralize most organic pollutants. The process allows flexibility to generate the hydroxyl radicals via variety of ways not limited to UV, H_2_O_2_/UV, O_3_/UV and H_2_O_2_/O_3_/UV etc. Despite the fact that it is good to treat the water for variety of pollutants, yet, this process adoption is limited as it was not cost-effective. For that reason, AOP is primarily used as pretreatment procedure to achieve biodegradability of waste water. Once biodegradability is achieved, the effluents then transferred to other processes that are cost-effective [104].

### 4.2 Ozonation

In this process, ozone is used to remediate the water contaminated with organic pollutants. This process occurs via two routes: 1) direct molecular ozone reactions 2) indirect pathways via ozone decomposition. Both process generates stable hydroxyl radicals which is subsequently used to remediate the water. When ozone reacts with organic pollutants, the end product are aldehydes and carboxylic acids. The aldehydes and carboxylic acid cannot be further decomposed using this process which is a serious limitation. Additionally, this process is highly pH-dependent, higher the pH efficient the process. The other limitation of this process is that it is very slow and selective in nature. Thus, this process has to be coupled with some catalyst to address the limitations mentioned above which is called as catalytic ozonation. Majority of catalytic ozonation process can be categorized as AOP [105].

### 4.3 Electrochemical methods

E2lectrochemical process generally involves oxidation reaction at the anode and reduction reaction at the cathode. The idea behind the use of electrochemical methods is to take advantage of both oxidation and reduction to remediate the pollutants in water. The oxidation process can be used to treat organic pollutants while the reduction process involves removal of heavy metal ions. The treatment of pollutants via electrochemical chemical oxidation can be performed by two routes: 1) direct anodic oxidation 2) indirect oxidation. Direct anodic oxidation occurs at the anode via direct charge transfer between pollutants and anode. The limitation of this route is requirement of prior adsorption of pollutants to the anodic surface. The indirect route involves generation of in-situ oxidant species at the electrode surface. The additional limitation of electrochemical oxidation route is it cannot be effectively used for remediation of water that contains chlorine-containing compounds such as organochlorine compounds, perchlorates and chlorates. Thus, electrochemical methods need to be combined with other methods as pre-treatment, post-treatment or as integrated treatment for complete removal of pollutants [106].

### 4.4 The photocatalytic

Remediation of water can be accomplished via photocatalytic oxidation (which has the most attention), or photocatalytic reduction. The photocatalytic remediation studies were conducted using a variety of metal oxides as catalysts. Metal oxides that have wide band gap provide characteristics such as non-toxicity and proven stability for water. Metal oxide nanoparticles are attractive materials to be investigated for water remediation. Metal oxides offer a high surface to volume ratio in nano-dimension thus facilitating the adsorption of pollutants to the catalyst surface. In particular, transition metal oxides exhibit superior photocatalytic activities for effective degradation of pollutants owing to its ability to control structural, crystalline, surface features. Titania suits above-mentioned properties hence it was widely investigated for the purpose of water remediation. In this review we will discuss recent developments in water remediation using titania as photocatalyst.

## 5. Applications of titania as photocatalyst

### 5.1 Doping of metals and non-metals in TiO_2_

The interest in using noble metals as dopants arises from the fact that it has superior ability to absorb visible light via localized surface plasmon resonance (LSPR) through collective oscillation of electrons in the surface. LSPR induce highly energetic electrons via absorption of light, which can be spread to neighbouring titania matrix which will significantly enhance the photocatalytic activity in the visible light region [107].

**Figure 4.**
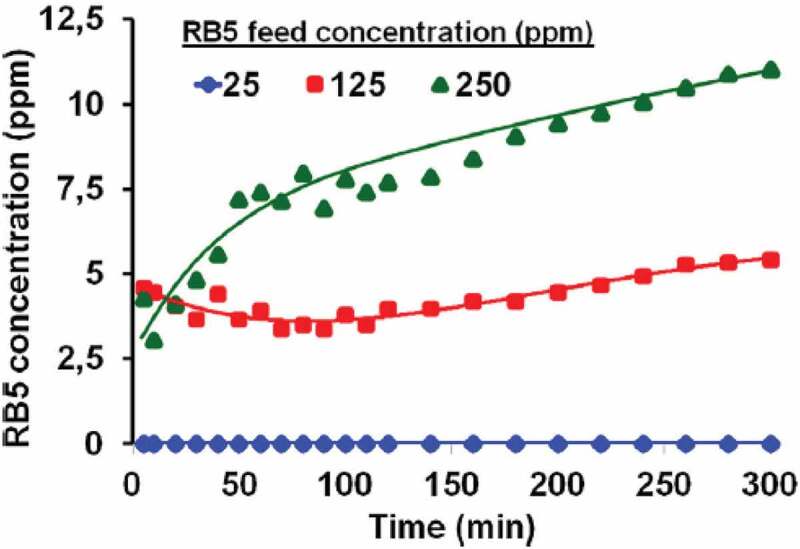
**Effect of flow rate on gold nanoparticle decorated titania for RB-5 dye degradation. Figure adapted from reference** [108]

Porous titania microbeads were synthesized Hamaloglu et al were decorated with gold or silver nanoparticles, which were used as catalyst in microfluidic reactor. Continuous photocatalytic dye degradation in a microfluidic system was demonstrated for dye degradation. Majority of photocatalytic degradation studies using titania photocatalyst are batch-type reaction. The authors of this study investigated the gold or silver decorated nanoparticles in microfluidic photocatalytic packed bed reactor. Bare titania microbeads and gold nanoparticle decorated titania microbeads were used to make slurry packed into fused silica capillaries with diameters ranging from 100 to 500 μm. Such prepared columns can be conveniently operable with HPLC columns with back pressures tolerable with HPLC pumps. Gold nanoparticle decorated titania showed the most promise for the RB5 dye degradation, at a flow rate of 5 mL/min. At a flow rate of 1 μL/min zero outlet dye concentration was observed for the above-mentioned photocatalyst (Figure 4). The results of that study were very encouraging to use this catalyst as pre-column or post-column in micro liquid chromatography applications for removal of coloured contaminants [108].

**Figure 5.**
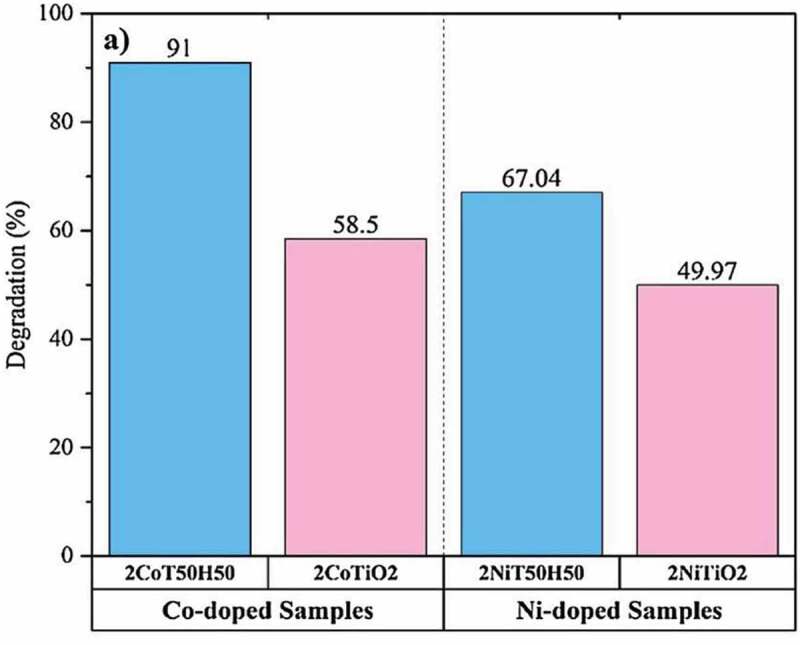
**Comparison of photocatalytic efficiency of Cu and Ni-doped titania. Figure adapted from reference** [109]

As mentioned in the introduction, dopants were investigated for the enhancement of the photocatalytic activity of titania. Co and Ni doping in titania improves the visible light absorption as the dopant can introduce impurity energy levels above the valence band and within mid-gap of titania. Salehi et al reported the synthesis of Co, Ni-doped titania and hydroxypatite doped titania along with Co and Ni were synthesized via sol-gel method for photodegradation of methylene blue [109]. Lower the concentration of dopant better the photocatalytic efficiency as higher concentration of dopants introduced agglomeration. The agglomeration problem were ameliorated by introduction of hydroxypatite, thus increasing the efficiency from 58.5% to 91% for Co-doped sample. Similar increase was observed Ni-doped hydroxypatite catalyst from 49.9% to 67% as shown in Figure 5. Better efficiency of hydroxypatite composite catalyst stems from better distribution of titania and its tendency to adsorb the organic molecules. Further, the doped composited titania exhibited 91.4% of original activity after its use in four cycles. Minimal drop in activity was due to blocking of active sites by dye molecules. The role of hydroxypatite was to impart ‘Adsorb and Shuttle’ role in the photocatalytic process.

Elmowafy and coworkers [110] reported the synthesis of titania with magnetic nanoparticles Fe_3_O_4_ on the reduced graphene oxide support. Coupling of titania with magnetic nanoparticles had exhibited higher photocatalytic activity for degradation of tartrazine dye than the individual titania or Fe_3_O_4_ magnetic nanoparticles alone loaded on graphene oxide. To put this in perspective the degradation with nanocomposite photocatalyst achieved 95% whereas for individual titania and Fe_3_O_4_ was 35% and 10%, respectively. Loading Fe_3_O_4_ nanoparticles along with titania facilitates easy separation of catalyst from solution through simple application of external magnetic field upon degradation is complete. The nanocomposite catalyst exhibited good recyclability with degradation efficiency of 90% after four cycles. The use of reduced graphene oxide as support offered increased adsorptivity of dye on photocatalyst, enhancement in visible light absorption through red shift arising from Ti-O-C and promotes the inhibition of charge recombination. The reduced graphene oxide acted as conductive layer to transfer the photogenerated electrons from the conduction band of magnetite to conduction band of titania and accumulated the hole in Fe_3_O_4_ thus increasing the charge separation.

**Figure 6.**
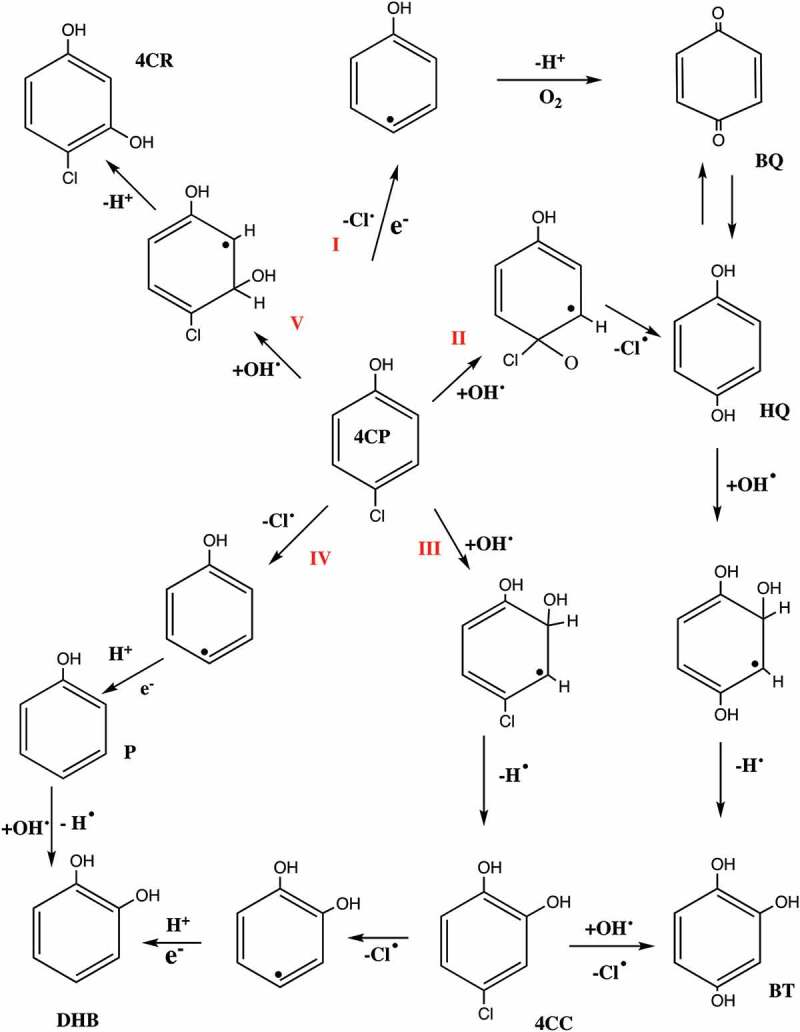
**Mechanism for degradation of 4-chlorophenol using W-Mo-TiO_2_** [111]

Garcia et al [111] employed dual dopant strategy to study the efficiency of titania photocatalyst. The dopants investigated were Mo and W. Catalysts were prepared with Mo-doped titania, W-doped titania and Mo-W-doped titania. The catalysts were synthesized using evaporation induced self assembly as it produces mesoporous structures with surface areas up to 191 m2/g. The catalysts were studied for degradation of 4-chlorophenol. The results were 46% more degradation for dual dopant titania than commercial degussa P25 catalyst. The dual dopant catalyst performed better for degradation due to synergistic effect of dual dopants that suppressed the recombination of photogenerated charges, enhanced radiation absorption capacity, large specific surface area and low crystallinity. Owing to tungsten presence as dopant had reduced the anatase band gap. The degradation of 4-chlorophenol involves quinones as main intermediates suggesting the mechanism via oxidation route as shown in Figure 6.

The band gap of titania can be altered by appropriate use of dopants. Cerium oxide is one such dopant to be used with titania to improve the efficiency as photocatalyst [112]. It has high capacity to store or release oxygen under redox conditions. Additionally, under reaction conditions, cerium can be reversible between +3 and +4 oxidation states that permits efficient electron transfer between cerium and titanium in the CeO_2_-TiO_2_ nanocomposite. The nanocomposite was synthesized using hydrothermal method and the as-synthesized nanocomposite was tested for photodegradation of common textile dye crystal violet and real industrial waste. Under optimal conditions, the nanocomposite exhibited 97% degradation of CV dye in 60 mins under visible light. The presence of ceria in nanocomposite allows faster transfer of the holes from titania to ceria. Such transfer facilitates increased generation of super oxide and hydroxyl radicals through synergistic effect, which contribute to overall activity of nanocomposite.

Thiruparanthagan et al [113] reported the synthesis (via sol-gel method) of titania impregnated with noble metals Au, Ag and Pt. Such prepared titania was subjected to degradation of tartrazine, Reactive Yellow-17 and Reactive Black-5 under UV and visible irradiation. The degradation of dyes using the above metal impregnated catalysts were found to be more effective under UV radiation than in the visible region. The degradation was adversely affected by inhibitors such as NaCl, Na_2_CO_3_ and ethanol whereas the electron acceptors such as hydrogen peroxide and potassium persulphate had facilitated degradation. Overall, the noble metals favored degradation of dye in the decreasing order of Au, Ag and Pt, respectively, and noble metal impregnated catalysts had superior activity than pure titania and commercial P-25. It was proposed by the researchers that Au had superior photocatalytic activity due to the small band gap value of Au-titania catalyst, which favours the visible light absorption. The superior activity of metal impregnated catalyst over pure titania was due to noble metals acting as electron traps, thus reducing the electron hole recombination. In a similar note, another study involved doping of Ag on titania-silica composite and titania-silica composites was used to degradation of various dyes such as Reactive Red 120, Acid Orange 20, Reactive Orange 16 and Congo Red 16 [114]. Acid Orange was the fastest to degrade and Congo red the least. The activity of titania-silica catalyst and Ag-titania-silica catalyst has improved activity than pure titania itself. Best performance was observed for Ag-titania-silica composite for all the chosen dyes. The superior performance was attributed to the nanocomposite’s low resistance and band gap value. Murcia et al [115] had improved the efficiency of titania catalyst by doping with Pt, along with it, they fluorized them to the commercial P25 and laboratory synthesized titania. The modification offered improvements in physicochemical and photocatalytic properties such as absorption of visible light, lower band gap values, increase surface areas etc. The modification was very particular based on structure of dye as it determines the adsorption on photocatalyst surface. Methylene blue and methyl orange was best degraded using laboratory synthesized titania that was fluorinated and Pt doped, however, for commercial titania that was just fluorinated had shown best degradation for aniline-based dyes. Arifin et al [116] studied copper ferrite as dopant instead of Cu and Fe separately. Copper ferrite has spinel type nanostructure that has narrow band gap was used in that study as dopant on titania. Further, it possesses direct band-gap that was positioned near the optimal value of sun spectrum and its conduction band that has high energy electrons with strong reducing power. Further, Cu and Fe has considered to be environmentally benign. Thus, the copper ferrite titania composite exhibited successful degradation of methylene blue dye.

**Figure 7.**
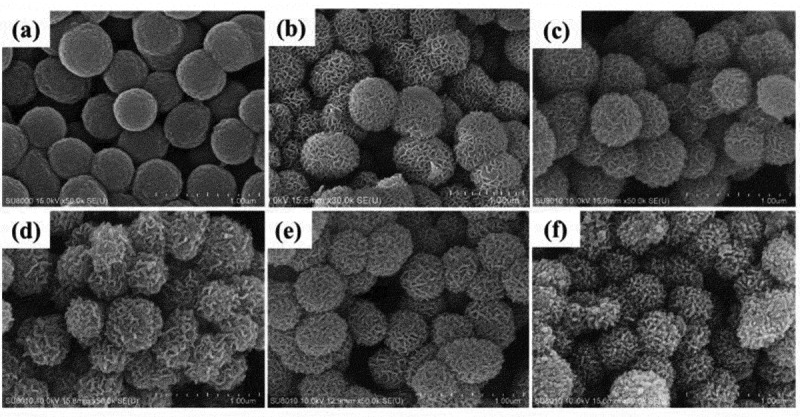
SEM images of a) titania precursor b) F-TiO_2_ c) Mn-FTiO_2_ d) Co-FTiO_2_ e) Ni-FTiO_2_ f) Cu-FTiO_2_ [117]

Nanomaterials in 3D hierarchical structures such as urchin-like and flower-like (F) offer novel catalytic properties owing to its large surface areas. Li et al [117] have combined the advantage of 3D hierarchical structure of titania with dopants to synthesize titania nanoflower with variety of transition metal (Mn^2+^, Co^2+^, Ni^2+^, and Cu^2+^) dopants (Figure 7). Such synthesized catalysts when tested for degradation of methylene blue dye in presence of hydrogen peroxide @30°C, metal-doped titania nanoflowers accomplished the degradation in 60–100 min, while just the control nanoflower titania had achieved only 35% degradation. Between the four dopants investigated Mn was found to be most efficient for dye degradation at low temperature. Kinetic studies revealed introduction of dopants lowered the activation energy of the oxidative degradation reaction. Additionally, all the metal-doped catalysts were robust even after 5 cycles.

Titania nanotubes doped with rare earth elements offers the opportunity to reduce the band gap of titania. La, Ce, Nd, Sm were discussed on that regards. Pr the lanthanide has the lowest band gap energy of 2.3 eV has the ability to absorb a broad solar spectrum owing to its physical and chemical properties. Thus, doping of Pr with titania offer bathochromic shift in optical absorption of catalyst. Ramya et al [118] synthesized Pr doped (up to 1 wt %) titania nanotube for degradation of Rhodamine B and Crystal violet (CV). The band gap of Pr doped titania kept decreasing from 3.08 eV to 2.99 eV and surface area of the catalyst decreased as well. Pr acts as electron trap and and facilitate the electron transfer to surface adsorbed oxygen to make super oxide radicals which is necessary for degradation of pollutants. The optimal loading of Pr was 0.4% and pH of 7 for efficient degradation of RhB and Crystal violet 90% and 93%, respectively, in the presence of solar light.

Traditional photocatalysis involves small catalyst particles which is a challenge to recover them after its use. To address this issue, membrane processes for separation of catalysts were reported in literature [119,120].^,^ However, membrane fouling after prolonged use is a challenge. Sheydaei et al [121] used ultrasonic radiation to limit fouling of membranes as well as improve the photocatalytic process that uses N-doped titania (P25) as catalyst. The micrometrical bubbles that form, grow and collapse due to ultrasonication causes hot spots with high temperature and pressure, facilitates mass transfer and chemical process efficiency. Membrane in this study was constructed using polyvinylidene fluoride. It was observed that power of visible light, presence of and inorganic oxidants (KIO_4_, H_2_O_2_ etc) favoured the degradation, increase in pH, radical scavengers adversely affected the degradation process. It was experimentally calculated that 23.51% of synergy existed between the sonolysis and photocatalytic process.

The limitation of titania is that it works best only under UV radiation. However, only about 5% is UV in the sunlight. Photosensitizers, dopants (metal and non-metal) and other semiconductors were used in conjunction with titania to utilize visible light effectively by extending its spectral response. Typically, one of the above-mentioned strategies were used in an investigation. Ramacharyalu et al used a dye sensitizer and a dopant as well to achieve enhancement in photocatalyst [122]. Thus, they tailored titania synthesized by hydrothermal method decorated with zinc and phthalocyanine and used it for degradation of malachite green and chemical warfare agent such as sulfur mustard. Degradation was achieved with sunlight exposure of as little as 60 min. But it was more than 6 h in the absence of zinc and phthalocyanine. This coupling has the potential to degrade several organic pollutants such as dyes and warfare agents along with use in dye-sensitized solar cells.

Mehrabi et al [123] synthesized titania-zinc titanate – αFe_2_O_3_ nanocomposite to decrease the band gap of titania from 3.2 eV to 2.17 eV thus extending the adsorption wavelength range to visible region thereby improving the photocatalytic activity. Along with reducing the band gap it produced more free hydroxyl radicals ultimately leading to a better oxidizing power of photocatalyst. Titania nanoparticles were first synthesized using sol-gel method followed by ZnTiO_3_ was synthesized on the surface of the titania. The resulting TiO_2_-ZnTiO_3_ was further modified with αFe_2_O_3_. Such synthesized catalyst was investigated for the degradation of both cationic and anionic dyes. The degradation efficiency of dye for the catalysts investigated in this study are as follows: TiO_2_ < TiO_2_-ZnTiO_3_ < TiO_2_-ZnTiO_3_-αFe_2_O_3_. ZnTiO_3_ and αFe_2_O_3_ had cubic structures that lead to an increase in the surface area of the composite leading to higher activity. Further, ZnTiO_3_ and αFe_2_O_3_ nanoparticles on the surface of titania acts as agents of electron capture, thus enhancing the separation and creating barriers for recombination of holes and electrons.

### 5.2 Silica supported titania

**Figure 8.**
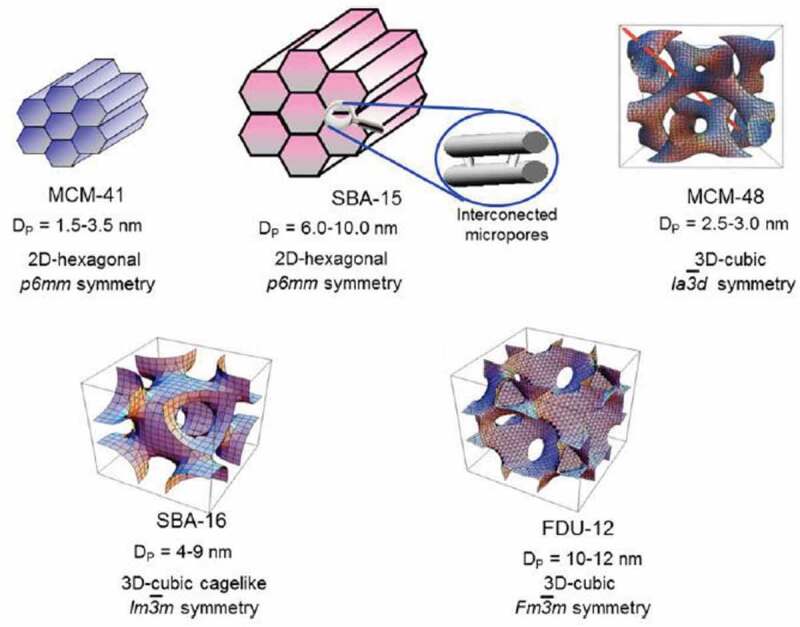
**Models of various mesoporous structures with pore diameters and symmetry. Figure adapted from reference** [131]

To increase the photocatalytic activity of titania, it should offer high surface area so it can offer more reactive sites. To achieve it, researchers had explored commercial silica [124], zeolites [125], clays [126], mesoporous silica (Figure 8) such as MCM-41, SBA-15 [127], KIT [128], activated carbon [129] etc. SBA-15 silica support has hexagonal pore structure with surface area as high as 1000 m2/g and thick pore walls can facilitate the efficient dispersion of titania and easy recovery of catalyst upon completion of reaction. Liou et al reported synthesis of titania incorporated SBA-15 via sol-gel synthesis for degradation of methylene blue dye [130]. The calcination temperature of the catalyst had influenced the photocatalytic activity. When the calcination temperature of catalyst increased from 300 to 600°C, photocatalytic activity increased. Subsequent increase in

Calcination does not affect the photocatalytic activity of catalyst. The catalysts had a uniform pore size distribution upto 600°C. The large pore size of SBA-15 allows efficient mass transfer of methylene blue dye hence improved activity towards dye degradation over the commercial silica gel. When titania is dispersed on silica, it offers the thermal, mechanical, and photocatalytic activity also it aids the formation of Ti-O-Si linkage. Durgadevi et al [132] reported the synthesis of titania on SBA-15 silica support, after which, they did the post-impregnation with CdS semiconductor nanoparticles because of its lower band gap. IR studies of the catalyst indicated the formation of Ti-O-Si bonds at broad absorption in 968 cm^−1^. The catalyst with various Ti/Si ratios were investigated for degradation of methylene blue. Superior photocatalytic activity was observed of Si/Ti of 0.5 when investigated without CdS. For the best catalyst with Si/Ti ratio of 0.1 and 0.2 wt % of CdS were deposited and further investigated for degradation of methylene blue. The optimal loading of CdS was 0.2 wt% had the best photocatalytic activity, any subsequent increase in CdS was found to affect the mesoporosity and surface area thereby reducing the activity of catalyst. Additionally, this catalyst was found to be effective for discolouration of industrial dye effluent as well in 90 mins.

### 5.3 Surface modification

**Figure 9.**
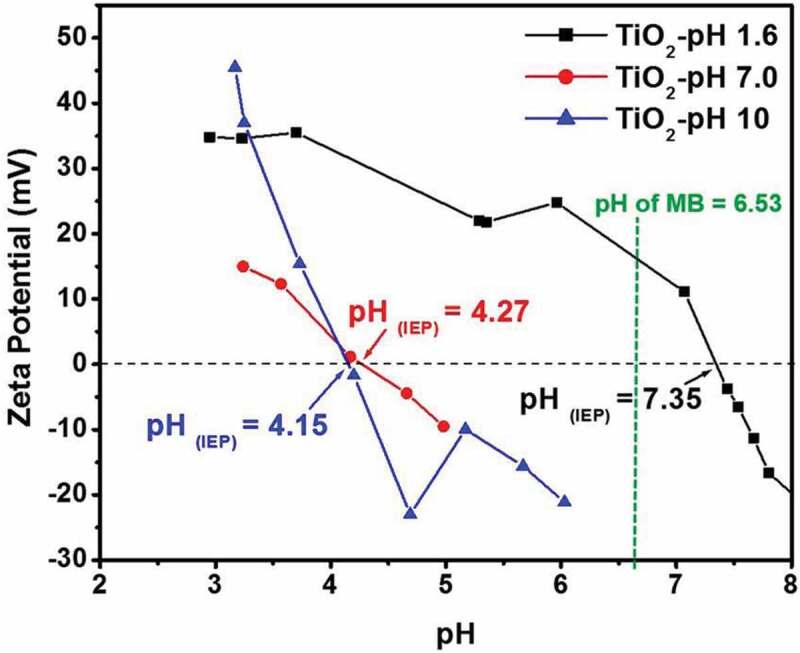
**Effect of pH on surface charge as measured by Zeta Potential studies. Figure adapted from reference** [133]

The pH of the solution influences the efficiency of the catalyst significantly. The surface characteristics, aggregated size of nanoparticles, charge of organic molecules influences the adsorptivity of catalyst. Surface doping, sensitization, creation of surface heterojunctions, using cocatalysts, enhancing surface area, use of surface F effects are some strategies to improve the surface charge properties of titania catalyst have been investigated. Azeez et al [133] have reported a simple approach for tailoring the surface charge of titania to improve the electrostatic attraction between nanoparticles. Titania nanoparticles synthesized under different pH conditions through hydrothermal sol-gel route affected the particle size, surface area and band gap. Higher the pH, the particle size of catalyst decreased thus increasing the surface area and limiting aggregation thus highest MB dye adsorption (at pH 10). At a pH less than 7, electrostatic repulsion due to positively charged titania was predominant. At pH 7 and 10 electrostatic attraction was predominant. Titania photocatalyst prepared at pH 1.6, 7 and 10 had point of zero charge 7.35, 4.27 and 4.15, respectively, thus higher pH has favoured the formation of negative charge on catalyst (Figure 9), further high pH facilitates scavenging of hydroxyl radicals faster, hence higher degradation efficiencies up to 97% was observed as methylene blue dye has a positive charge.

Nanocrystalline titania that has surface charges opposite to the environmental pollutants offers electrostatic force of attraction for efficient removal of dyes. Low-temperature synthesis of nanocrystalline titania near neutral surface charge was prepared by sol-gel method followed by base extraction with a final pH of 6 and 9. The nanocrystalline titania synthesized under pH 9 has better adsorption capability for anionic dyes methyl orange and indigo carmine because of its higher negative surface charge. Around 10 wt % titania the titania catalyst synthesized with pH 9 has highest selectivity for methylene blue degradation, whereas, at pH of 6, selective degradation of indigo carmine was observed over methyl orange in the aqueous mixture.

Murcia et al [134] had applied the sensitization strategy to increase the efficiency of titania photocatalyst in dye degradation. The sensitizers are typically a chromophore that improves the visible light absorption of photocatalyst. The chromophores are generally anchored to the semiconductor surface to achieve the enhancement in visible light absorption. Such chromophore molecules absorb visible light and excite electrons, and these electrons get transferred to the conduction band in the semiconductor, leading to decrease of its band gap value. Titania powder and titania nanotubes were synthesized and modified by Quinizarin and Zinc protoporphyrin. Such synthesized catalysts were tested for dye degradation of methyl orange. For traditional titania synthesized with Quinizarin sensitizer performed the best due to better substrate–catalyst interaction an important factor. However for titania nanotubes Zinc protoporphyrin sensitized titania nanotube had superior performance than Quinizarin sensitized nanotube which was attributed to high surface area. Additionally, the sensitization in nanotubes brought electron confinement in photocatalyst. Such confinement decreases the electron-hole recombination in comparison to titania nanopowders that has high surface area than nanotubes. On the downside, for nanotubes, titania also degraded the sensitized dye.

**Figure 10.**
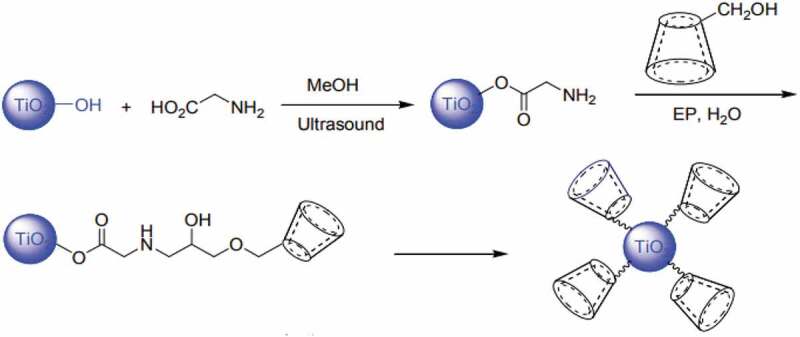
**Scheme for a diagram for the binding of β-cyclodextrin onto TiO_2_ nanoparticles** [135]

Mohammadi et al [135] had investigated β-Cyclodextrin and glycine to modify the surface of titania to improve the photocatalytic efficiency of dye degradation. β-Cyclodextrin has cone-shaped hydrophobic inner cavity that has nonpolar microenvironment to host several organic and inorganic pollutant molecules via formation of inclusion complexes. Thus, β-Cyclodextrin was used to modify (see Figure 10) the surface of titania to improve the adsorption of pollutants thereby increasing the photocatalytic activity of titania catalyst. Further, the modified catalyst with cyclodextrin has superior activity due to multiple hydroxyl groups in the inner cores of hydrophobic cavity. Also, the catalyst was good to degrade the cationic and anionic dyes too. In another study by Masilompone et al had used chitosan-lignin-titania nanocomposite for the removal of brilliant black dye from aqueous solution [136]. This nanocomposite offers improved sorption capacity due to oxygen functional groups on the three components, the amine groups in chitosan and the pi-electron system in the benzene motifs of lignin matrix. The chitosan-lignin serve as nanoadsorbent. The nanocomposite was found to be efficient under acidic conditions for the removal of dye due to protonation of amine groups.

Salama et al [137] had modified the surface of P25 with amino functionalization followed by crosslinking with cellulose acetate – carbon nanotube to form nanocomposite fibers. Such synthesized nanofibers were investigated for degradation of methylene blue and indigo carmine dye. The degradation efficiency was observed to be lower at high dye concentrations for both the dyes due to saturation of active catalytic sites. Highest degradation efficiency for both dyes were observed at pH 2 as it may enhance the electrostatic interactions between the catalyst and dye. Also, the catalyst required a very low power intensity of 40 W at 353 K. The full degradation took 180 mins for indigo carmine and 300 mins for methylene blue dye. The nanocomposite had exhibited preference for indigo carmine over methylene blue for degradation.

### 5.4 Miscellaneous strategies using titania

While titania photocatalyst was widely used for environmental remediation, sometimes, synthesis of the titania photocatalyst itself may not be green in nature. Saikumari et al had explored green tea extract as sol-gel template to synthesize the titania photocatalyst in a soft non-toxic route [138]. The choice of green tea extract was due to the richness of polyphenols which can serve as reducing and capping agent, further, the active components (phenolic compounds, proteins, carbohydrates, lipids and vitamins C and E) of green tea can facilitate the growth of titania crystals (less than 25 nm average particle size) during the sol-gel process. That catalyst was found to be effective for complete degradation of congo red dye under solar irradiation and it possesses very high recyclability (up to five cycles) with only marginal decrease in activity.

Titania nanotubes (TNT) was investigated for its potential to superior electrical and optical properties via high specific surface area. The improved photocatalytic efficiency was attributed to tubular morphology and wider bandgap under UV irradiation. TNT synthesis was reported by Ali et al [139] via rapid breakdown anodization method using chloride and perchlorate ions in an electrolyte where metallic titanium was converted to TNT in seconds after the application of voltage. Interestingly, undoped TNTs prepared by rapid breakdown anodization was efficient for discolouration of dyes (cationic and anionic) under sunlight than in UV light due to its mechanism of degradation (for Rhodamine B). Annealing temperature of TNT dictates the types of phases. The as-synthesized TNTs had a band gap of 3.04 eV and it got shifted towards the visible region (2.88 eV) as it got annealed. Further, annealing reduced the electron-hole recombination. At high annealing temperature, surface area decrease was offset by favourable crystal structure and decreased electron-hole recombination.

While most of the studies in literature for use of titania as photocatalyst was primarily devoted to degradation of dyes, it was also investigated for degradation of antibiotics in water system. Belhouchet et al investigated the use of calcite coated titania as photocatalyst for degradation of tetracycline a common antibiotic in aquaculture [140]. The treatment of pollutants involved advanced oxidation process with hybrid catalysts of titania with natural materials such as sepiolite, montmorillonite, diatomite, kaolinite, attapulgite etc. Calcite which is environmentally benign, was coated to titania prepared via sol-gel method. At 30% loading of calcite in titania exhibited the best degradation of tetracycline under neutral conditions. Iron doped titania was studied by Tsiampalis for sulfamethaxoazole degradation under solar radiation [141]. Moosavi et al reported the degradation of Amoxicillin using supported titania nano photocatalysts [142].

Metformin an antidiabetic drug on of the common pharmaceutical pollutants typically found in hospital and domestic wastewater which has a profound effect on human health even at low concentrations. Nezar et al [143] had investigated degradation of metformin using Degussa catalyst combined with hydrogen peroxide an electron acceptor to improve the efficiency by trapping the electrons. The degradation of metformin was performed by sunlight irradiation.

Common downside in the photocatalytic process for removal of pollutants in wastewater is incomplete recovery of the photocatalyst itself, which will lead to secondary pollution. To address this concerns immobilization of photocatalyst on supports such as silica, glass and carbon-based materials. Hydrogels are also investigated for the purpose of immobilization. Hydrogels are 3-D crosslinked hydrophilic polymer that can swell and trap water in its network. Gum tragacanth [144] a natural biopolymer that was investigated for its multifaceted roles as an emulsifier, thickener and binder in several industries which has water soluble part that is neutral, and insoluble part which is due to methoxylated acids with the ability to swell. Rahimdhokt et al [144] used gum tragacanth hydrogel to immobilize titania photocatalyst to remove methylene blue dye from wastewater. They found under optimal conditions the waste water was decolourized by up to 89%. Similar to this approach cellulose biopolymer was used as support to immobilize the titania by Cardenas and coworkers [145]. The hydrophilic nature of cellulose pose a challenge to interact with hydrophobic pollutants. This shortcoming can be addressed by chemical modification, in this instance, simply dipping the titania cellulose nanocomposite dipping in nylon 6 solution in formic acid. Nylon 6 stabilizes the photocatalytic nanoparticles and effective isolation of pollutant from water through a variety of mechanisms such as dispersion forces and H-bonding. Such synthesized catalyst had better photocatalytic performance for dye degradation (methyl orange) under UV and sunlight for short times of irradiation ranging from 20 to 40 mins.

Titania inverse opals that are constructed by colloidal template self-assembly, contains periodic structures has unique refractive index periodicity leading to unique photonic properties. The porous structure has tunable macro and mesopores. Thus titania inverse opals when coupled with carbon nanomaterials improves photon capture and photogenerated charge separation ensue a highly active photocatalyst. Diamantapoulou et al [146] reported synthesis of inverse opals via evaporation induced coassembly were loaded with graphene oxide nanosheets. Despite the fact that loading of graphene oxide nanosheets decreased the mesoporosity, however, the surface functionalization enhancement compensated the increased adsorption of pollutants (methylene blue). At 664 nm, slow light propagation of photons in titania skeleton accelerated the dye degradation kinetics. At higher loading of nanographene oxide, superior degradation of dye was observed due to slow photon effects, adsorption and interfacial charge transfer. When nanographene oxide was loaded in conventional P25 catalyst, there was a drop in degradation of photocatalytic activity was observed due to mesopore clogging.

**Figure 11.**
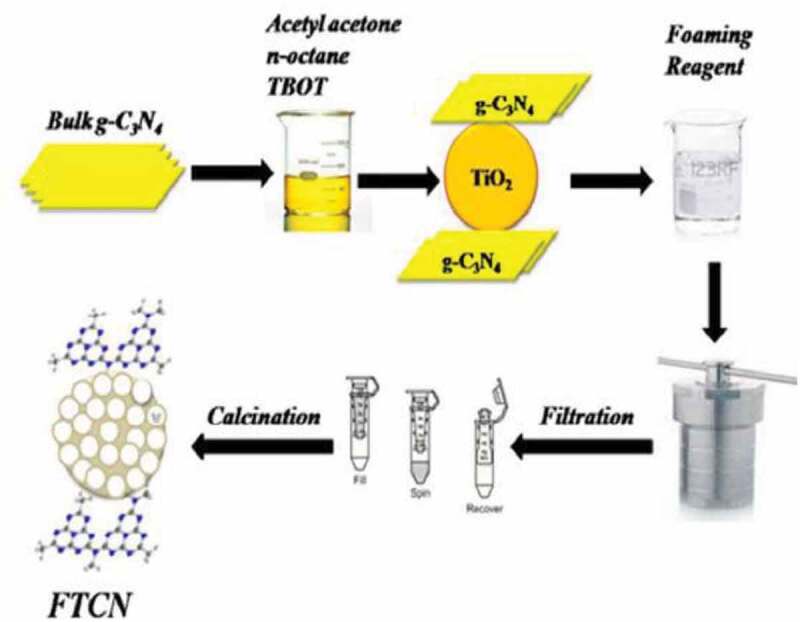
**Scheme for synthesis of foamed titania graphitic carbon nitride** [147]

Graphitic carbon nitride (g-C_3_N_4_) had recently gained interest due to its responsiveness to visible light in catalysis for its stability to light and charge transfer capabilities. It has a band gap of 2.7 eV. When g-C_3_N_4_ was combined with titania, it enhances visible light absorption and offer space for adsorption of dyes and acts as photoactive charge carrier. Thus, foamed titania carbon nitride nanocomposite was synthesized by micro emulsification method as shown in Figure 11 [147]. When the synthesized catalyst was evaluated for degradation of methylene blue and Rhodamine B dye, at 9% loading of titania, the degradation was 95.5 and 93%, respectively, under visible light in as short as 30 and 50 min, respectively. The enhancement in photodegradation was attributed to its excellent surface morphology, high surface areas that offer sufficient contact with dyes and closed interfaces. In the nanocomposite investigated in this study the electrons from the conduction band were shifted to interface between g-C_3_N_4_ and titania which effectively decreased the recombination and the trapped electrons reacted with dissolved oxygen to produce superoxide radicals. Meanwhile, the holes in the valence band are captured by hydroxyl radical or water molecules to decompose the dyes.

The efficiency of a photocatalyst also affected by its support, as the support offers high contact surface area which is crucial for pollutants to interact with active catalyst. For immobilization of catalyst, aluminum stainless steel, ceramics, quartz and sand are primarily investigated. Another familiar support is fiberglass strips which offers a simple and quick route. Stainless steel woven wire mesh is a good substrate owing to its large surface area and the possibility of light to penetrate through it. Oblak et al [148] studied stainless steel mesh and hemmed knitted fibreglass strips as supports for titania immobilization (P25, P90 and PC500). For the three different types of titania, P90 exhibited best adhesion by sonication test with 17% loss of photocatalyst. Horizontal dip-coating worked the best for fibreglass strips and electrophoretic deposition for stainless steel mesh. Both supports were promising for phenol degradation (upto 94%) and Reactive Blue 19 dye (upto 99%).

Titania was immobilized on supported glass carbon hollow fibres through two-stage rapid thermal processing to prevent the release of titania in environment [149]. Titania hollow fibres has high surface area to volume ratio and sponge-like porous inner structure that can create a reactor with increasing selectivity and efficiency through combination of photocatalysis and phase separation process together. The rapid thermal processing involves partial carbonization of polymer binder (1-methyl-2-pyrrolidinone) with short heating duration at a constant temperature around 1073 K. Further, the presence of carbon decreases sintering of titania and the subsequent phase transformation. Additionally, the carbonaceous char brings mechanical strength and microporosity as a structural support. The titania investigated in this study was P25 which on immobilization on carbon hollow fibres had a mechanical strength as high as 11 MPa and surface area of 218 m2/g. Further, it exhibited an improved photocatalytic activity in UV light than the control titania for the removal of acid orange 7 dye.

If a certain material is black, it may possess the ability to absorb energy from visible light and IR radiation as well. With that in mind, several researchers tried to manipulate the color of titania to enhance the light-absorbing properties. Black titania was first reported by Chen et al [150], followed by several research groups reported that material and used it in traditional applications of titania. Followed by that, blue [151], brown [152], and red titania [153] were reported. Ullattil et al reported low-temperature synthesis of defective brown titania porous flower aggregates using Mn(II) as reducing agent and template for broad and longer wavelength absorbing material via sol-solvothermal process [154]. The synthesized brown titania had flower like aggregates that contains ample defects states where titania was found as trivalent ion and oxygen vacancies. Those defects created excess surface charges behave as reaction centre for photocatalysis. The black and yellow titania had an average crystallite size of 6 nm which offers good redox power due to quantum effect. The brown and yellow titania had surface positive charge; hence it was selective in degradation negatively charged dye such as methyl orange.

Bio-inspired fabrication of black titania nanocomposites that has moth-eye-like nanostructures were reported by Liu et al for solar-driven clean water generation via solar steam generation and photocatalytic degradation [155]. The moth-eye-like black titania nanorod arrays were immobilized on carbon cloth, has hierarchical nanostructures cause light to be trapped in the nanorod gaps, leading to several internal reflections until it gets completely absorbed. This enhances the light absorption and minimizes heat loss. The black titania nanocomposite in moth-eye-like form introduces oxygen vacancies and surface disorder which aids in excellent light absorption in full solar spectrum range and offers active sites for photocatalytic reaction and minimizes recombination of photogenerated electrons and holes. Further, the carbon cloth used in the nanocomposite was conductive to the transfer of electrons. Black titania nanocomposite exhibited RhB degradation of 95% was achieved in 100 min of irradiation under solar illumination of 1 kWm^−2^.

## 6. Environmental concerns and recyclability

We had discussed thus far the benefits of titania in its nanosize on how it can help clean up the aquatic environment polluted by dyes and antibiotics etc. Before we wrap up it is important to acknowledge the hazards posed by the nanosized titania itself to the environment as much as any other nanosize material. The catalysts we discussed in the review is not 100% efficient from leaching thus it may end up in the environment. In addition to the leaching from catalysts, titania nanomaterial has the potential to pollute from the use of certain day-to-day materials such as cosmetics, paints, food industry, pharmaceuticals, agricultural industry in the form of fertilizers and pesticides and construction materials [156–163]. The toxicity of nanomaterials is very dependent on size and surface chemistry of nanoparticles in water. The aggregation state and size of titania dictates the extent of toxicity. The aggregation of nano titania in turn were influenced by size, shape of particles, pH and ionic strength [164]. The toxicity of titania arises from the fact that size is similar to cellular components and proteins, which facilitates the travel inside the human body. Such entered titania can cause DNA and pulmonary damages via production of reactive oxygen species (ROS), oxidative stress (OS) and inflation in the vasculature and lungs [165–170]. During the process of OS, the production of ROS overwhelms the defense for antioxidants by the cell hence the adverse sequence arises. Also, OS generates the superoxide anion, hydrogen peroxide, hydroxyl radicals and peroxynitrites via ROS [171]. These reactive oxygen species cause oxidative DNA damage through increased levels of cellular nitric oxide [172–174]. With this in mind, future research on titania-based catalysts should focus on design that mitigates the leaching of titania into an environment that might harm our ecosystem. If leaching occurs, the scientific community should focus on ways to recover it from the environment efficiently.

## 7. Conclusions

To conclude, this review summarizes literature about the generic importance of titania nanocomposites that arise from its superior photocatalytic properties followed by its generic synthesis strategies found in the literature to synthesize them. Finally, we discussed the recent advances in the synthesis of titania catalyst ranging from its synthesis strategies to achieve the enhancements to suppress the electron-hole recombination. In general, it was observed that the advancements in the synthesis and manipulation of catalyst lead to efficient removal of toxic organic pollutants primarily dyes. Additionally, it was explored for removal of pesticides and medicines. Overall, titania catalyst was found to convert all the organic pollutants to environmentally benign by-products. The studies presented in this review represent the enhancements of titania catalyst either through reduction in electron-hole recombination or increasing the absorption of light extending to visible region. The advancements in this field are promising to present opportunities to combat a variety of pressing needs of humankind and help in thriving our ecosystem.
